# The Implementation of Social Media in Neurosurgery: A Systematic Review of the Literature

**DOI:** 10.1007/s00701-025-06695-1

**Published:** 2025-10-18

**Authors:** Elisa Colombo, Lara Maria Höbner, Valerie Blom, Inka Berglar, Aron Alakmeh, Daniel de Wilde, Victor Gabriel El-Hajj, Luca Regli, Carlo Serra, Victor E. Staartjes, Gustav Burström

**Affiliations:** 1 Machine Intelligence in Clinical Neuroscience & Microsurgical Neuroanatomy (MICN) LaboratoryDepartment of NeurosurgeryClinical Neuroscience Center, University Hospital Zurich, University of Zurich, Zurich, Switzerland; 2 Department of Clinical Neuroscience, Karolinska Institutet, Stockholm, Sweden; 3 Capio Spine Center Stockholm, Löwenströmska Hospital, Stockholm, Sweden

**Keywords:** Social media, Social networking, Neurosurgery, Education, Publishing

## Abstract

**Background:**

The advent of social media has significantly transformed various medical specialties, including neurosurgery. A systematic review of the literature was conducted to characterize the utilization of social media in neurosurgery and to evaluate the impact of social media usage in neurosurgery. Furthermore, the study aimed to determine the demographics of social media users in neurosurgery and delineate their purposes for engaging with social media platforms.

**Methods:**

A comprehensive literature search was conducted across the PubMed, EMBASE, Scopus, and Cochrane databases to identify studies investigating the role of social media in neurosurgery. Articles were screened for relevance, and selected studies were systematically reviewed and analyzed to assess the integration of social media within neurosurgical practices.

**Results:**

105 studies were included. 2023 represented the year with the most published articles (28%). Most studies (52%) addressed general neurosurgery, followed by intracranial (24%) and spine surgery (24%). X (formerly Twitter) was the most frequently studied platform (46%), followed by YouTube (38%) and Facebook (30%). The primary purposes of social media use were patient education (36%), evaluation of the impact (22%), healthcare provider education (20%), collaboration (9%), research dissemination (8%), and career development (6%). 64% of studies targeted healthcare professionals, while 36% focused on patients. Sentiment towards social media use was positive in 50% of studies, negative in 19%, and neutral or exploratory in 31%.

**Conclusion:**

The literature highlights a notable increase in the use of social media in the neurosurgical field, particularly for education, impact analysis and research distribution. Platforms like X have become central for academic exchange and professional networking. Having a social media presence can be beneficial for neurosurgeons and can positively impact patient reviews, the department’s standing, and may even contribute to academic success. Furthermore, social media facilitates interdisciplinary collaboration and access to educational content.

**Supplementary Information:**

The online version contains supplementary material available at 10.1007/s00701-025-06695-1.

## Introduction

The digital revolution has fundamentally changed communication across all professional fields, including the field of medicine[82], thanks to the introduction of new tools and platforms. Social media platforms, defined by the *Cambridge English Dictionary* as websites and computer programs that allow people to communicate and share information on the internet using a computer or mobile phone, particularly stand out for their widespread adoption and impact[24, 31, 55]. They can be used for various purposes, including communication, networking, marketing, and information dissemination[15].

In the medical field, the use of social media platforms is equally diverse. Individual practitioners, medical institutions, and scientific journals use these platforms to share the latest advances in clinical research, facilitate real-time discussions, and promote interdisciplinary exchange. Simultaneously, important medical knowledge is made easily accessible to the broad public. Key aspects of social media platforms, such as user-friendly interfaces, effortless navigation, and widespread accessibility, have led them to become an important aspect of modern healthcare[68].

In neurosurgery, a highly specialized and continuously evolving field, social media may serve as a valuable vehicle for information exchange, professional networking, and patient outreach. In 2019, approximately 70% of neurosurgical attendings, residents, and fellows used social media for professional purposes[91, 119]. Of note, platform usage varies considerably depending on the user group.

Patients tend to prefer platforms such as Facebook or X (formerly Twitter), while neurosurgeons are more likely to use Doximity or Linkedin[103]. Furthermore, social media engagement is more prevalent in private practice caregivers compared to neurosurgeons practicing in academic institutions[103], and usage is mostly limited to younger individuals[8, 103]. Despite its considerable potential, the increasing use of social media in neurosurgery also presents several challenges. These include risks of patient privacy violation, the propagation of unrealistic expectations, an overemphasis on imaging at the expense of clinical context, and concerns regarding the reliability and accuracy[5].

All things taken into consideration, the benefits of social media may be increasingly significant in the field of neurosurgery. However, despite its growing influence, the role of social media in the neurosurgical field has not been thoroughly analyzed. Considering both the promising potential and its affiliated challenges and risks, it is crucial to critically assess the role of social media in neurosurgery. Therefore, this systematic review aims to explore its function within the discipline and provide guidance for patients, students, caregivers, faculty, and journals in evaluating the advantages and disadvantages of integrating social media into neurosurgery. Furthermore, it aims to facilitate the development and understanding of influential content and enhance the interaction between patients and neurosurgeons.

## Methods

### Overview

A systematic review was conducted to identify studies investigating the implementation of social media within the neurosurgical field. The review was performed in accordance with the Preferred Reporting Items for Systematic Review and Meta-Analyses (PRISMA) guidelines[88]. A completed PRISMA checklist is available in Supplementary File 1.

### Search Strategy

The PubMed MEDLINE, Embase (Elsevier), Scopus (Elsevier), and Cochrane Library databases were queried using the following search query:

("neurosurg*" OR “neurological surger*”) AND ("social media" OR "media exposure" OR “youtube” OR "twitter" OR "facebook" OR "tiktok" OR "linkedin" OR "instagram" OR "snapchat").

All references were retrieved on October 21 st, 2024, and imported into a web-based systematic review platform (Rayyan.ai) for deduplication and subsequent screening[87]. Title and abstract screening, full-text review, and final study inclusion were independently conducted by two reviewers (V.B. and E.C.). Any disagreements were resolved through discussion and consensus, and if consensus could not be reached, a third independent reviewer (V.E.S.) was consulted.

### Selection Criteria

Studies were eligible for inclusion if they explicitly addressed the role, impact, or use of social media in any neurosurgical subspecialty, with a focus on professional or academic applications. All age groups, sexes, and ethnicities were considered. Articles were required to be published in English, German, Italian, or Dutch. Exclusion criteria included: (1) studies not directly related to neurosurgical practice, (2) studies focused exclusively on other medical specialties, and (3) article types such as case reports, commentaries, and letters to the editor.

### Data Extraction

Data extraction was independently performed by two reviewers (V.B. and E.C.) using a standardized form. Extracted variables included year of publication, journal, neurosurgical subspecialty, social media platforms assessed, purpose of use, target audience, and sentiment. Sentiment was evaluated based on the narrative tone of the conclusions of the included studies.

### Data Analysis

A narrative synthesis following the Synthesis Without Meta-analysis (SWiM) guidelines was performed[21]. Data were presented as counts and percentages. All descriptive statistical analyses were conducted using Excel (Version 2408, Microsoft, Redmond, Washington, USA) and R statistical software (version 4.2.3., R Foundation for Statistical Computing, Vienna, Austria).

## Results

### Search Results

**Fig. 1:**
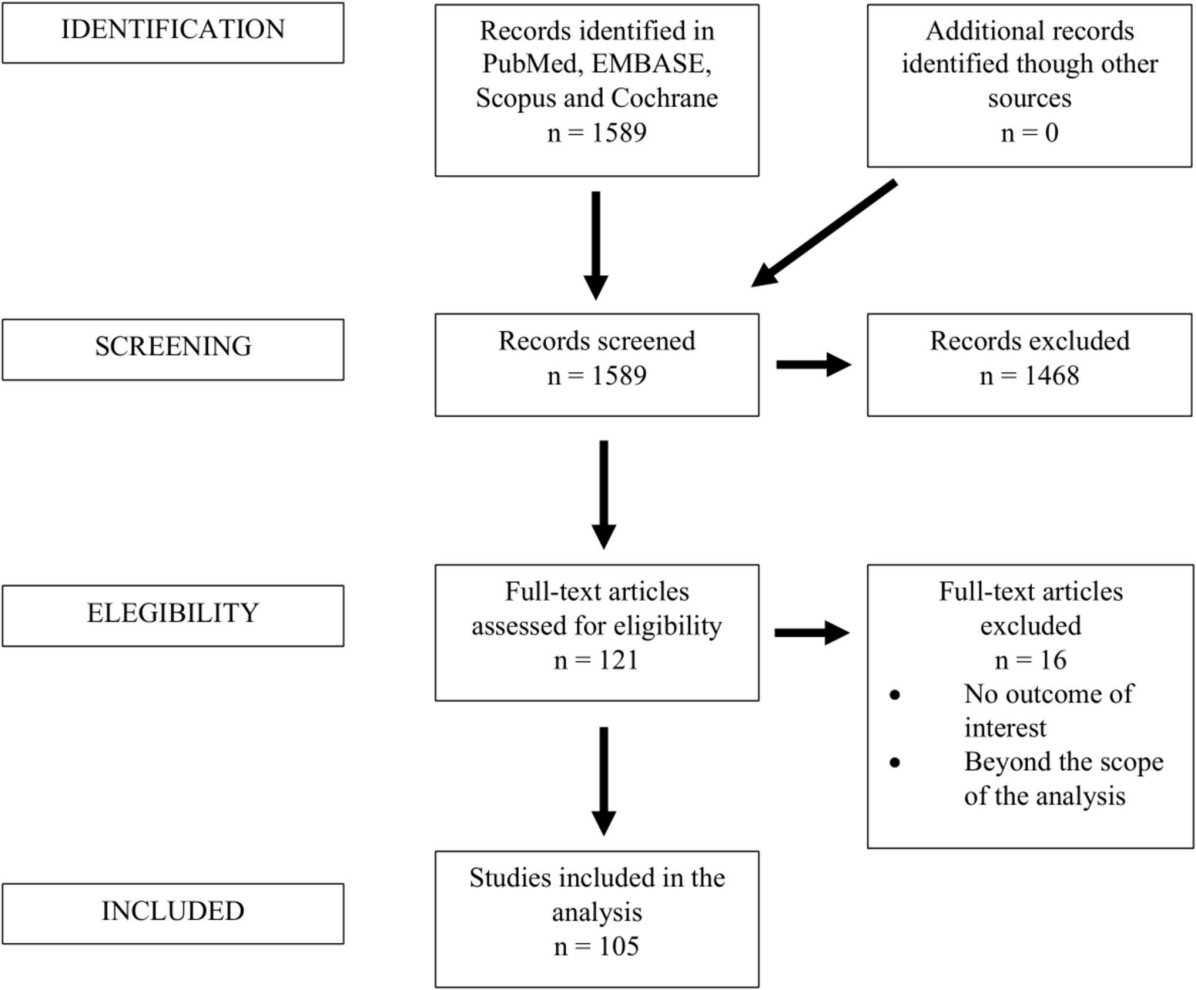
PRISMA flow diagram illustrating the study selection process

A total of 1,589 unique records were identified across databases. Following title and abstract screening, 121 studies underwent full-text review, of which 105 met the inclusion criteria[1, 2, 4, 6–14, 17–20, 22, 23, 25–30, 32–38, 40–54, 56, 57, 59–67, 69, 71–81, 83–86, 89–95, 97–101, 104–106, 108–124]. The study selection process is illustrated in the PRISMA flowchart (Fig. 1), and an overview of study characteristics is summarized in Table 1.

**Table 1:** Overview of General Characteristics of Included Studies

Authors	Year of publication	Subspecialty	Topic of article	Audience	Primary Aim	Social Media Platforms Used/Investigated
Abdullah M et al	2024	General NCH	Neurosurgery and Social Media: A Bibliometric Analysis	Neurosurgeons	Collaboration	Web of Science database
Agarwal N et al	2021	Spine	Scoliosis correction surgery	Patients	Patient information	Instagram
Alamri A et al	2018	General NCH	Brainbook	Patients	Collaboration	Twitter, YouTube and Instagram
Alotaibi NM et al	2017	Brain	Brain Aneurysms and Subarachnoid Hemorrhage	Patients	Patient information	Facebook, Twitter, YouTube
Alotaibi NM et al	2016	General NCH	Possible factors for engagement and reach among SM users in neurosurgery	Neurosurgeons	Impact of social media	Facebook, Twitter
Alotaibi NM et al	2016	General NCH	Relationship betw. Social media metrics and academic indices	Neurosurgeons	Impact of social media	Facebook, Twitter
Alotaibi NM et al	2016	General NCH	Social media in NCH	Neurosurgeons	Impact of social media	Facebook, Twitter, YouTube
Arfaie S	2022	General NCH	Canadian Medical Student Interest Group in Neurosurgery (CaMSIGN)	Neurosurgeons	Collaboration	Youtube
Arpa A et al	2023	Spine	Spina Bifida	Patients	Education—Patient	YouTube
Aydin SO et al	2023	Spine	Spinal cord stimulation	Neurosurgeons	Education—Doctors	Instagram
Baker JD et al	2021	Spine	Cervical Fusion	Patients	Education—Patient	Youtube
Bandyopadhyay S et al	2022	General NCH	Women in NCH	Neurosurgeons	Career advancement	SoMe
BernsteinDN et al	2021	Spine	Spine surgeons SM use	Neurosurgeons	Impact of social media	Facebook, Twitter, Instagram, LinkedIn, Youtube
Bird C et al	2020	Brain	Tumor	Patients	Impact of social media	Facebook pages and groups, YouTube videos, and Twitter or Instagram accounts
Boudreau HS et al	2023	General NCH	Neurosurgery residency websites (NRWs)	Neurosurgeons	Education—Doctors	Neurosurgery residency websites (NRWs)
Bozkurt I et al	2021	General NCH	Social media in NCH	Neurosurgeons	Impact of social media	SoMe
Canty MJ et al	2019	General NCH	Selective dorsal rhizotomy for children	Patients	Patient information	Facebook, Twitter, YouTube
Chaurasia B et al	2022	General NCH	Neurosurgery Cocktail	Neurosurgeons	Education—Doctors	Facebook
Kumar et al	2019	General NCH	NCH operating videos	Neurosurgeons	Collaboration	YouTube
Chisolm P et al	2021	General NCH	Residency program applications and Social Media	Neurosurgeons	Education—Doctors	Twitter, Facebook, Instagram, residency websites, and the Visiting Student Application Service (VSAS)
Cloney MB et al	2023	General NCH	Social media in NCH	Neurosurgeons	Impact of social media	Twitter, Instagram, Facebook, Doximity Residency rankings, US News & World Report rankings
Conti A et al	2023	General NCH	Neurosurgery Cocktail	Neurosurgeons	Impact of social media	Facebook, Twitter, YouTube, Instagram, blogs, Reddit
Daggubati LC et al	2021	General NCH	NCH and Twitter	Neurosurgeons	Impact of social media	Twitter
D'Amico RS et al	2021	General NCH	BRAINterns	Neurosurgeons	Education—Doctors	Open-access webinar series ("BRAINterns")
Davies B et al	2018	Spine	Degenerative Cervical Myelopathy	Patients	Patient information	Facebook, Twitter, Google AdWords
Doenges JE et al	2020	General NCH	Carotid endarterectomy (CEA)	Neurosurgeons	Education—Doctors	Youtube
Donnally CJ et al	2018	Spine	Physician rating	Neurosurgeons	Career advancement	Facebook, Twitter, Instagram, Healthgrades, Vitals, Google
Donnally CJ et al	2020	Spine	Physician rating	Neurosurgeons	Career advancement	Facebook, Twitter, Instagram, Healthgrades, Vitals, Google
Efe IE et al	2024	Brain	Awake craniotomy	Patients	Education—Patient	Youtube
El Naamani K et al	2023	General NCH	Neurosurgery Influencers on Twitter	Neurosurgeons	Collaboration	Twitter
Elarjani T et al	2022	General NCH	US NCH training programs and Social Media	Neurosurgeons	Impact of social media	Twitter
Elkaim LM et al	2020	Brain	Deep brain stimulation	Patients	Patient information	Twitter
Encarnacion Ramirez MJ et al	2024	General NCH	NCH among Low-Middle income countries	Neurosurgeons	Education—Doctors	Twitter, Facebook, Instagram and LinkedIn
Faisal UH et al	2023	Brain	Cerebrovascular Q&A webinar series	Neurosurgeons	Education—Doctors	Zoom, YouTube
Gajjar A et al	2023	Brain	MoyaMoya	Patients	Patient information	Twitter, TikTok, Instagram
Gajjar AA et al	2023	Brain	Cerebral aneurysms	Patients	Patient information	Twitter, TikTok, Instagram
Gajjar AA et al	2024	Brain	Cerebral cavernous malformations	Patients	Patient information	Twitter and Instagram
Garcia G et al	2024	General NCH	Social events, Networking	Neurosurgeons	Career advancement	X
Gill AS et al	2021	Brain	Pituitary and Endoscopic Skull Base Surgery	Patients	Education—Patient	Youtube, Google
Giordano M et al	2024	Brain	Vestibular schwannoma	Patients	Education—Patient	Youtube
Godolias P et al	2022	Spine	Lateral Spine Surgery	Neurosurgeons	Education—Doctors	YouTube
Haider AS et al	2023	Brain	Tumor	Neurosurgeons	Patient information	Facebook, Twitter, Instagram, LinkedIn
Hamidi N et al	2021	Brain	Tumor	Patients	Patient information	Facebook, Twitter, YouTube
Heisinger S et al	2023	Spine	TLIF	Patients	Patient information	Youtube
Holderread B et al	2022	Spine	Anterior Cervical Discectomy	Patients	Patient information	Instagram
Hussein A et al	2024	Spine	The Virtual Global Spine Conference (VGSC)	Neurosurgeons	Education—Doctors	Facebook, Twitter, Youtube
Jenkin Sy J et al	2023	Brain	Hydrocephalus	Patients	Education—Patient	Youtube
Joshi SS et al	2018	General NCH	Whats app as referral service	Neurosurgeons	Impact of social media	Whatsapp
Kearns K et al	2023	General NCH	J of Neurooncology and their social media activity	Neurosurgeons	Visibility of research	Journal of Neurooncology, Facebook, Twitter
Kim C et al	2018	General NCH	Neurosurgeons digital profiles	Neurosurgeons	Career advancement	Twitter.com, Doximity.com, Linkedin.com, YouTube.com, Facebook.com,
Knopf JD et al	2020	General NCH	Surgery videos	Neurosurgeons	Education—Doctors	Youtube, other websites
Korkmaz M et al	2024	Spine	Unilateral biportal endoscopic spine surgery	Neurosurgeons	Education—Doctors	Youtube
Kozyrev DA et al	2023	General NCH	Whats app	Neurosurgeons	Impact of social media	Whats app
Krakowiak M et al	2021	Brain	Arteriovenous malformations	Neurosurgeons	Education—Doctors	Youtube
Lamano JB et al	2021	General NCH	Physician rating	Neurosurgeons	Patient information	Twitter, Facebook, YouTube, Instagram
Lavadi RS et al	2022	General NCH	Productivity of neurosurgeons using SM	Neurosurgeons	Collaboration	SoMe
LE CH et al	2024	General NCH	Neurosurgery Cocktail	Neurosurgeons	Collaboration	Facebook, Twitter, Instagram, LinkedIn
Levett JJ et al	2023	Spine	Pediatric spine surgery	Patients	Patient information	Twitter
Linzey JR, et al	2020	General NCH	The Journal of Neurosurgery Publishing Group (JNSPG) social media team (SMT)	Neurosurgeons	Impact of social media	Facebook, Twitter
Lynch CP et al	2022	Spine	Cervical spine literature and Social Media	Neurosurgeons	Visibility of research	Twitter, Facebook, News
Madhugiri VS et al	2024	General NCH	General NCH	Neurosurgeons	Impact of social media	Medline indexed neurosurgical journals
Malhotra K et al	2023	General NCH	Neurosurgery Awareness Month	Neurosurgeons	Collaboration	4 social media (Twitter) assessment tools (Sprout Social, SocioViz, Sentiment Viz, and Symplur) and Google Trends
Mata-Gomez J et al	2018	General NCH	NCH and Social Media in Spain	Neurosurgeons	Impact of social media	Facebook, Twitter, Youtube
Mayo I et al	2019	Brain	External Ventricular Drain (EVD)	Neurosurgeons	Education—Doctors	Youtube
McAlpine H et al	2021	Brain	Primary brain tumors	Patients	Patient information	SoMe
McBriar JD et al	2022	General NCH	NCH and TikTok	Patients	Patient information	TikTok
Moens M et al	2024	Spine	Spinal Cord Stimulation	Patients	Education—Patient	Facebook, Twitter, YouTube, Instagram, blogs, Reddit, Vimeo, Google
Mohile NV et al	2023	Spine	Lumbar Disc Herniations	Patients	Education—Patient	YouTube
Mota Telles JP et al	2020	General NCH	NCH and Hashtags	Neurosurgeons	Collaboration	Twitter, Instagram
Newall N et al	2021	General NCH	Case-based discussions (CbDs)	Neurosurgeons	Education—Doctors	Twitter
Niazi F et al	2023	Brain	Microvascular decompression	Patients	Patient information	Twitter
Nouri A et al	2022	General NCH	NCH and Social Media	Neurosurgeons	Impact of social media	SoMe
Ochuba P et al	2021	General NCH	Microsurgical techniques	Neurosurgeons	Education—Doctors	Youtube
Pando A et al	2023	General NCH	Neurosurgeons SM print	Neurosurgeons	Impact of social media	Instagram
Patel MR et al	2022	Spine	Media presence and dissemination of spine literature	Neurosurgeons	Visibility of research	Twitter, Instagram
Phillips HW et al	2019	General NCH	Use of social media amongst neurosurgeons	Neurosurgeons	Impact of social media	SoMe
Powell K et al	2023	General NCH	Financial conflict of interest	Neurosurgeons	Impact of social media	Twitter
Raturi V et al	2024	General NCH	Most influential women in NCH	Neurosurgeons	Impact of social media	Twitter
Riccio I et al	2023	General NCH	Concerns about SM by neurosurgeons	Neurosurgeons	Impact of social media	Twitter
Richardson MA et al	2021	Spine	Online attention and AAS in seven spine journals.#	Neurosurgeons	Visibility of research	Twitter, Facebook
Sader N et al	2020	Brain	Endoscopic third ventriculostomy and endoscopic third ventriculostomy with choroid plexus cauterization for pediatric hydrocephalus	Patients	Impact of social media	YouTube
Safa A et al	2023	Spine	Robotic Spine Surgery	Patients	Education—Patient	YouTube
Sama A et al	2020	Spine	Physician rating	Neurosurgeons	Career advancement	Facebook, Twitter, Instagram, Healthgrades, Vitals, Google
Samuel N et al	2017	General NCH	NCH and Youtube	Neurosurgeons	Patient information	Youtube
Sciscent BY et al	2023	General NCH	Adoption of Twitter by matched neurosurgery applicants before and during the COVID-19 pandemic	Neurosurgeons	Education—Doctors	Twitter
Sledzinska et al	2021	Brain	Meningioma Treatment	Neurosurgeons	Education—Doctors	Youtube
Sorba EL et al	2023	General NCH	Social media on altmetrics	Neurosurgeons	Visibility of research	Twitter and European Journal of Neurosurgery
Sotudeh H et al	2018	General NCH	Gender difference	Neurosurgeons	Visibility of research	Facebook, Linkedin,
Stogowski P et al	2022	Spine	ALIF	Patients	Education—Patient	Youtube
Subramanian T et al	2023	Spine	NCH Spine and TikTok	Patients	Patient information	TikTok
Swiatek PR et al	2023	Spine	Anterior Cervical Discectomy and Fusion (ACDF) Surgery	Patients	Patient information	Social media outlet
Szmuda T et al	2021	Brain	Aneurysms	Patients	Patient information	YouTube
Szmuda T et al	2020	Brain	Hydrocephalus	Patients	Education—Patient	Youtube
Temel MH et al	2024	Brain	Traumatic Brain Injury	Neurosurgeons	Education—Doctors	Youtube
Teton ZE et al	2020	General NCH	The Neurosurgical Atlas	Neurosurgeons	Education—Doctors	Facebook,Twitter, Instagram, Neurosurgical atlas website and app
To J et al	2023	Spine	Spinal cord conditions and Reddit	Patients	Patient information	Reddit
To J et al	2023	Brain	Cranial neurosurgical disease	Patients	Patient information	Reddit
To J et al	2024	General NCH	NCH and Reddit	Patients	Patient information	Reddit
Tripathi SD et al	2022	Brain	Brain tumors	Patients	Patient information	Reddit
Udawatta M et al	2019	General NCH	Use of social media amongst neurosurgeons	Neurosurgeons	Impact of social media	SoMe
Wang J et al	2017	General NCH	Altmetric	Neurosurgeons	Visibility of research	Facebook, Twitter, blogs, and mainstream media sources
Waqas M et al	2021	General NCH	NCH residents and Social Media	Neurosurgeons	Visibility of research	SoMe
Ward M et al	2019	General NCH	Educational quality of NCH Resources on Youtube	Neurosurgeons	Education—Doctors	Youtube
Ward MS et al	2023	Brain	Brain tumor	Patients	Patient information	Youtube
Yakar F et al	2020	General NCH	NCH and Instagram	Patients	Patient information	Instagram

### Publication Characteristics

**Fig. 2:**
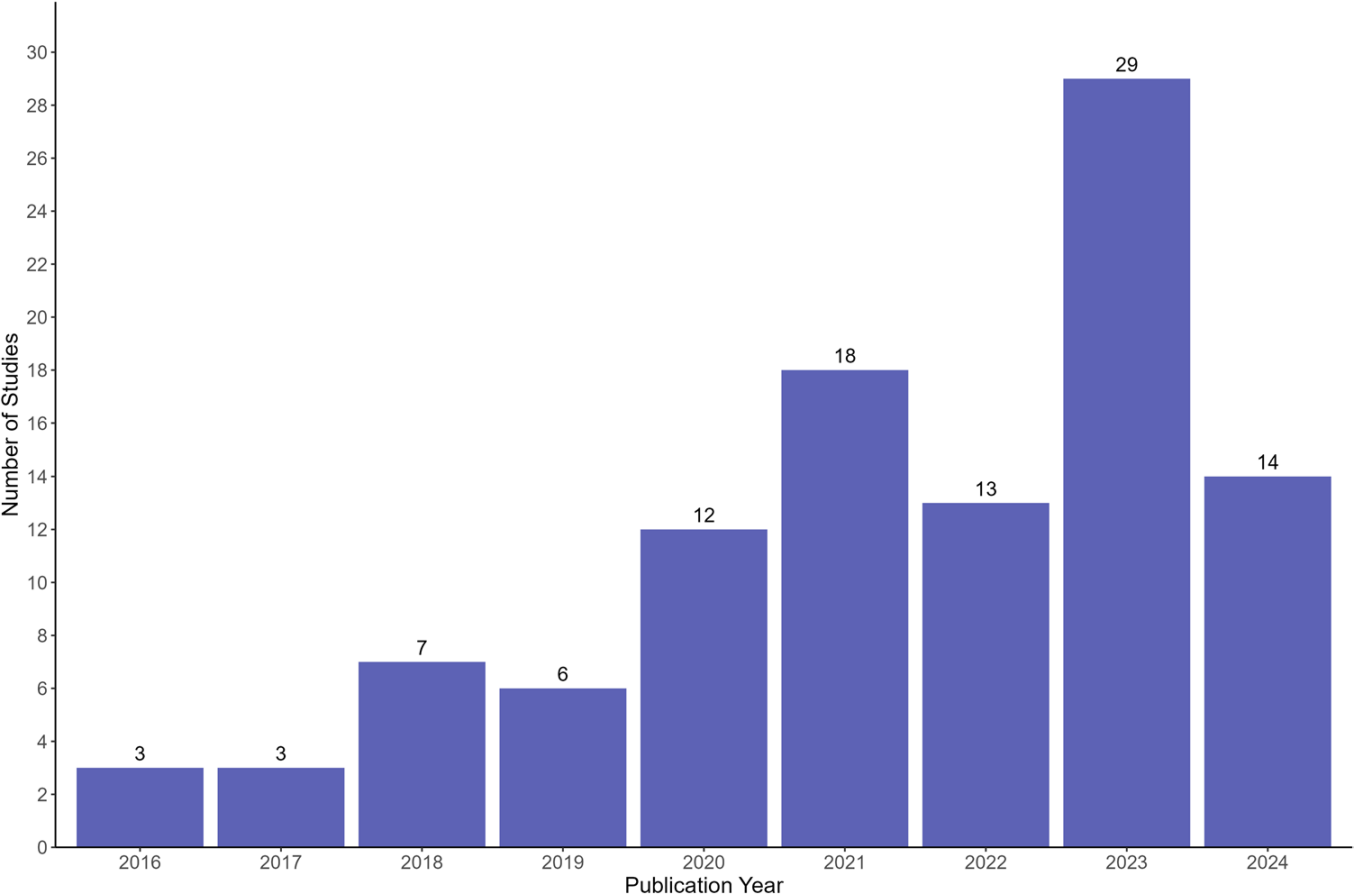
Bar chart of included studies by publication year

The included studies were published between 2016 and 2023, with a marked increase in publications over the past eight years (Fig. 2). The year 2023 accounted for the highest number of studies, with 29 studies (28%) being published in that year. *World Neurosurgery* and its companion journal, *World Neurosurgery X,* were the most common publication venues, collectively accounting for 39% (n = 41) of all included studies.

### Neurosurgical Subspecialties

Of the 105 studies, 55 (52%) addressed general neurosurgical topics without focusing on a neurosurgical subspecialty. 25 studies (24%) focused on the use of social media in the context of intracranial surgery, and 25 studies (24%) examined its implementation in spine surgery.

### Social Media Platforms Investigated

X (formerly known as Twitter) was the most frequently investigated platform (n = 49, 47%), followed by YouTube (n = 42, 40%), other platforms (n = 34, 32%), Facebook (n = 32, 30%), Instagram (n = 26, 25%), LinkedIn (n = 7, 7%), Reddit (n = 6, 6%), and TikTok (n = 4, 4%) (Table 2). Among all included studies, more than half (n = 56, 53%) assessed multiple social media platforms.

**Table 2:** Social Media Platforms Investigated Across Included Studies

Platform	Number of Studies	Year of Highest Prominence
X (formerly Twitter)	49 (25%)	2023
YouTube	42 (21%)	2021
Facebook	32 (16%)	2020
Instagram	26 (13%)	2023
LinkedIn	7 (4%)	2024
Reddit	6 (3%)	2023
TikTok	4 (2%)	2023
Other	34 (17%)	2023
**Total**	**200**	

### Primary Aims of Social Media Use

#### Patient Information and Education

**Fig. 3:**
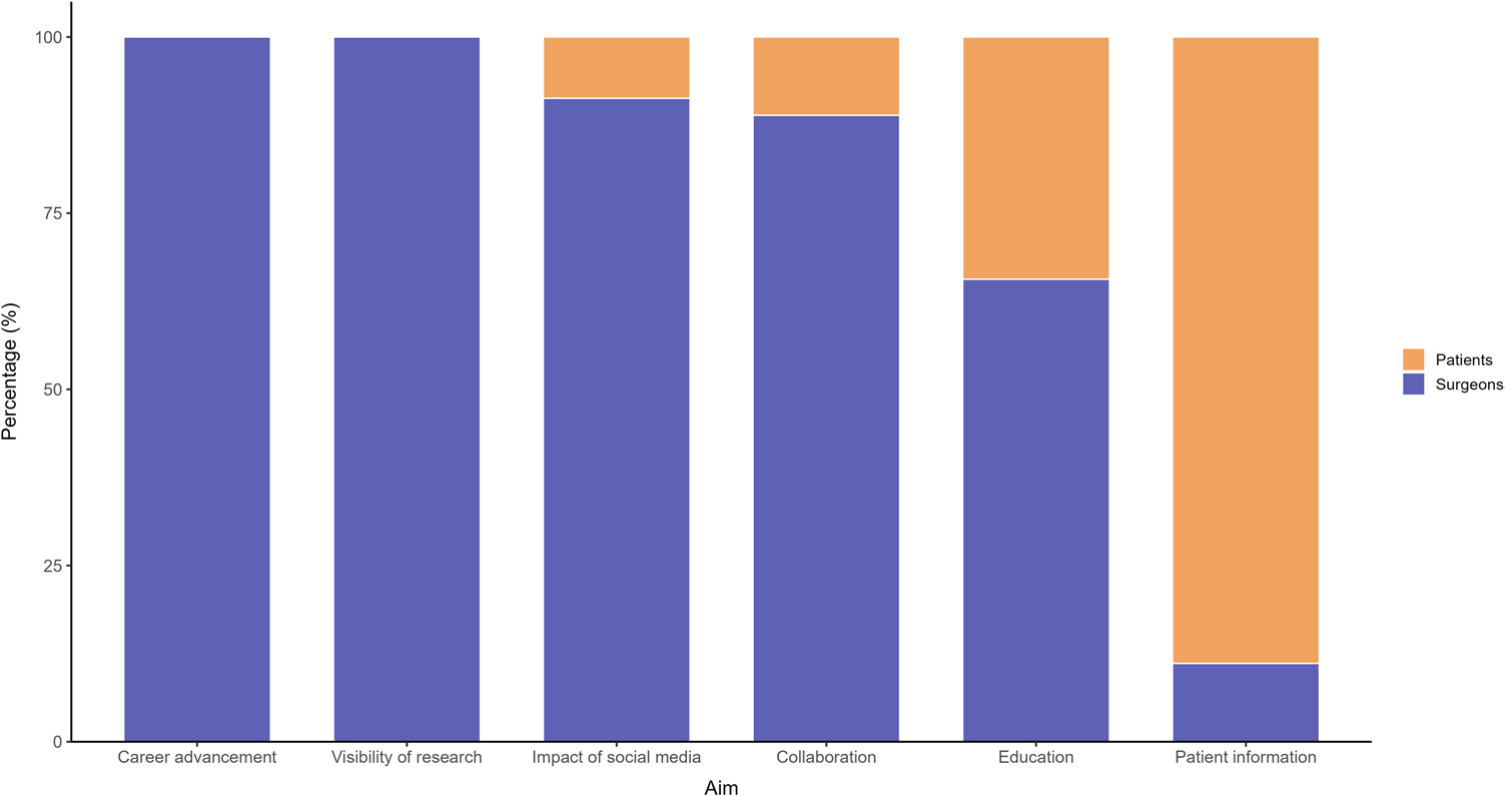
Stacked bar chart illustrating the primary aims of included studies, categorized by target group

The most frequently reported purpose (n = 38, 36%) of social media use in neurosurgery was patient education and information dissemination. These studies highlighted the role of social media in providing clear, accessible content aimed at countering misinformation, improving health literacy, and supporting informed decision-making. An overview of the study categorizations is presented in Table 3 and Fig. 3.

**Table 3:** Distribution of Included Studies by Primary Aim and Platform Evaluated

Primary Aim	Number of Studies	X (formerly Twitter)	YouTube	Facebook	Instagram	LinkedIn	Reddit	TikTok	Other
Patient information	27	12	9	6	8	1	4	4	3
Impact of social media	23	14	7	10	6	2	1	0	11
Education—Doctors	21	6	11	5	4	1	0	0	7
Education—Patient	11	1	11	1	1	0	1	0	2
Collaboration	9	5	3	1	3	1	0	0	3
Career advancement	6	5	1	4	3	1	0	0	3
Visibility of research	8	6	0	5	1	1	0	0	5
	**105**	**49**	**42**	**32**	**26**	**7**	**6**	**4**	**34**

The total number of studies is lower than the sum of platform-specific counts, as several studies evaluated multiple social media platforms simultaneously

#### Evaluation of Social Media Impact

23 studies (22%) evaluated the broader impact of social media within the field of neurosurgery. These studies noted benefits such as enhanced education, professional networking, and outreach, while also reporting risks such as the spread of misinformation and ethical concerns. Quantitative measures, including follower counts, impressions, and interaction metrics, were commonly used to characterize social media reach and usage patterns.

#### Education of Healthcare Providers

21 studies (20%) addressed the use of social media as an educational tool for neurosurgeons and trainees. These studies reported the use of social media platforms to deliver educational content via formats such as video lectures, webinars, and live-streamed procedures. They also described peer-to-peer interactions facilitating the exchange of clinical insights, procedural techniques, and treatment strategies. Furthermore, the use of social media platforms as a means to share curated resources such as clinical guidelines and recent publications was noted.

#### Collaboration

Nine studies (9%) investigated the role of social media in fostering collaboration among neurosurgeons and other healthcare professionals. Reported uses included consultations on complex cases across institutional and geographic boundaries, interdisciplinary engagement for joint research or clinical care, and the organization of virtual events such as conferences, workshops, and webinars. These initiatives were noted to enhance accessibility and participation, particularly in resource-limited settings.

#### Research Visibility

Eight studies (8%) explored the use of social media in enhancing the visibility of neurosurgical research. These studies described how platforms were used to disseminate findings beyond traditional academic audiences, promote open-access materials, and encourage academic discourse.

#### Career Advancement

Six studies (6%) examined the role of social media in professional growth and career advancement for neurosurgeons. These studies emphasized the value of platforms in building professional identity and reputation by showcasing expertise, sharing achievements, and increasing visibility within the neurosurgical community and broader medical landscape.

### Target Audiences

67 studies (64%) focused on neurosurgeons as a primary target audience, with topics such as continuing education, peer networking, and professional development. The remaining 38 studies (36%) targeted patients, primarily focusing on the delivery of health-related information and support for informed decision-making.

### Sentiment Analysis

**Fig. 4:**
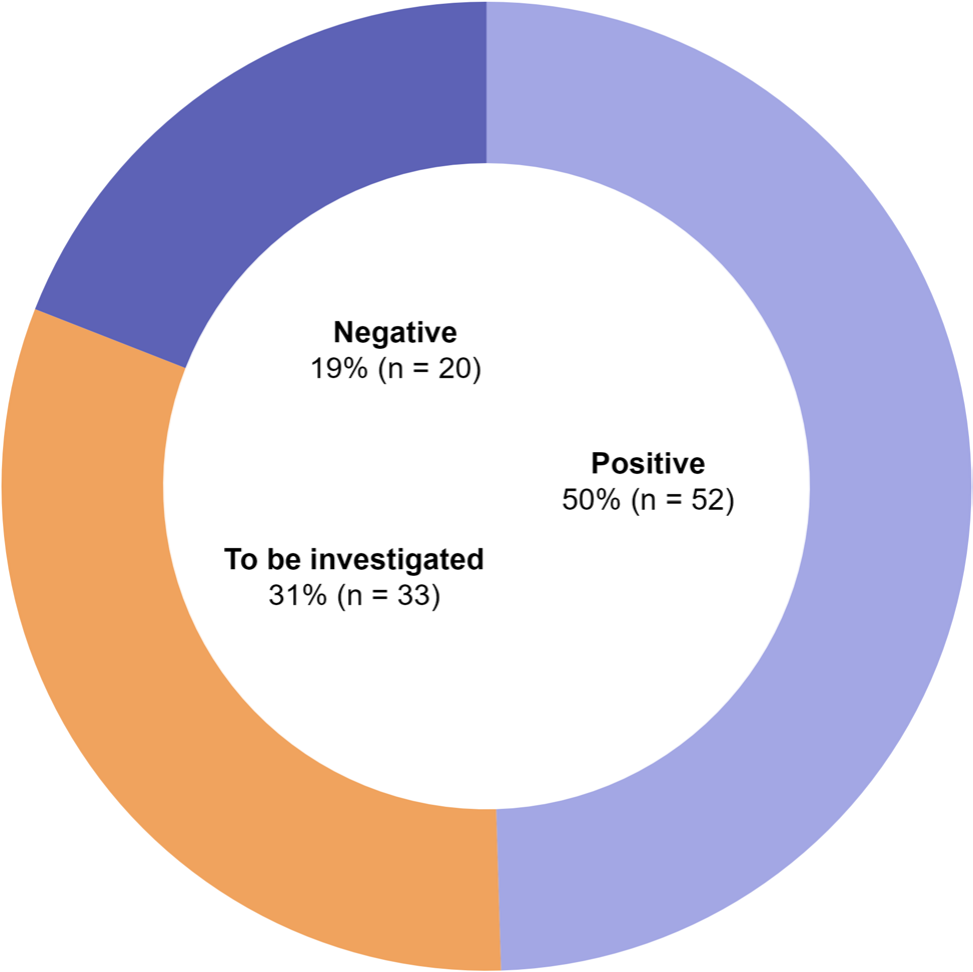
Donut chart depicting the distribution of sentiment across included studies

Sentiment toward social media use in neurosurgery was described as predominantly positive in 52 studies (50%). 20 studies (19%) conveyed predominantly negative sentiment, most often related to concerns about misinformation, data privacy, and ethical considerations. 33 studies (31%) indicated that further investigation was needed to better characterize the effectiveness and risks associated with social media use in neurosurgery (Fig. 4).

## Discussion

The integration of social media into the field of neurosurgery represents a significant shift in how neurosurgeons, patients, and the broader medical community interact, communicate, and share knowledge[27]. This systematic review highlights the implementation and integration of social media platforms into the field of neurosurgery from 2016 to the present time, underscoring both its potential benefits and the inherent challenges that accompany this integration.

**Fig. 5:**
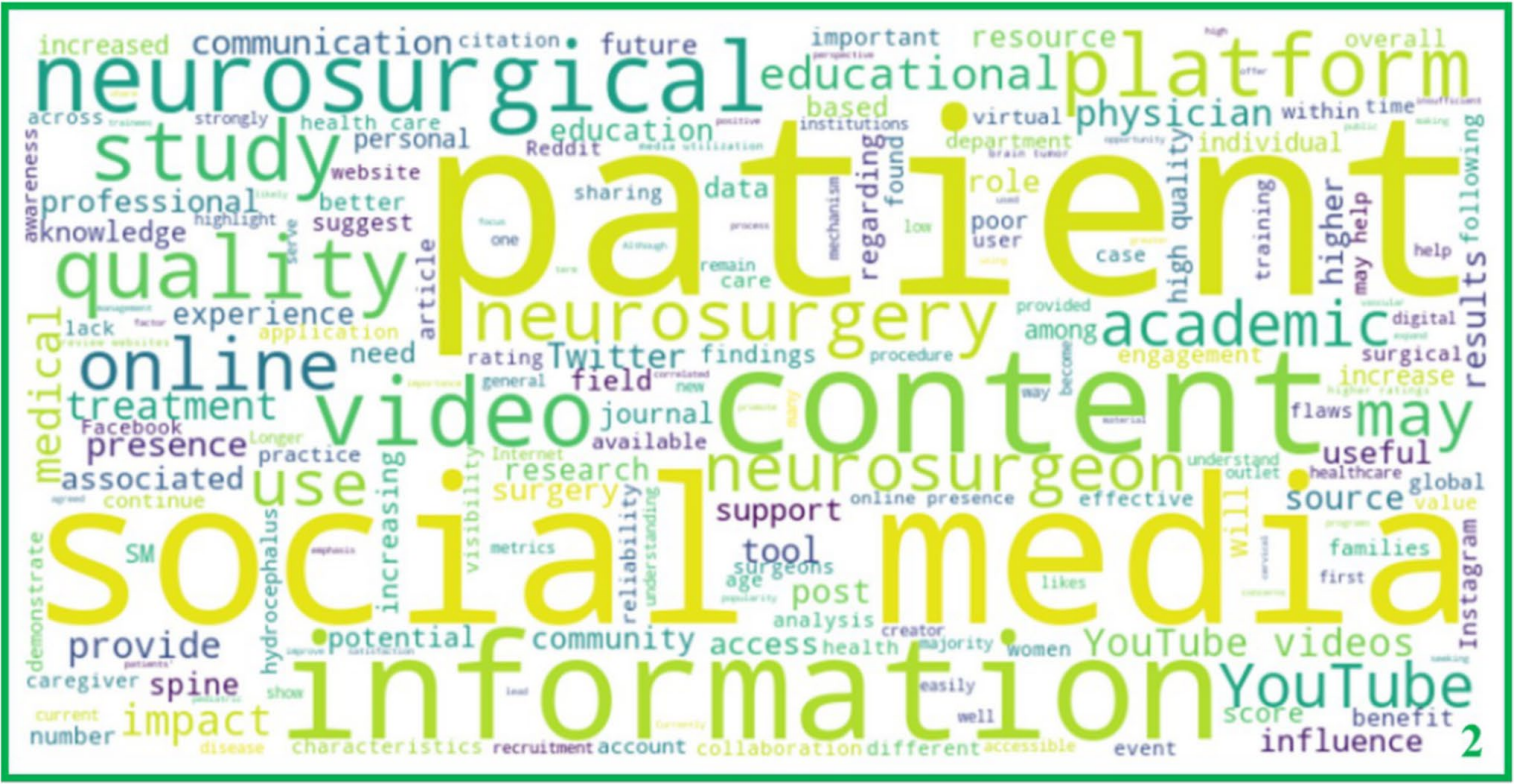
Word cloud of the most frequently occurring terms in the included studies

The reviewed literature demonstrates a significant rise in the use of social media by neurosurgeons and neurosurgical organizations, underscoring its potential in patient and healthcare provider education and research dissemination (Fig. 5). Among the social media platforms, X (formerly Twitter) and Instagram have emerged as the predominant channels for sharing academic insights and building professional networks in the neurosurgical field. As a result, the majority of professionally produced neurosurgical content is concentrated on those two platforms, reflecting a broader trend seen in most medical and scientific fields[24, 55]. In contrast, video content on YouTube, while abundant, is generally of low to moderate quality[51, 54, 108]. Authors have thus suggested some form of quality-control/peer-review, especially for patient education, which may translate into a curated video bank for neurosurgical pathologies[48, 64]. Similarly, platforms such as TikTok, though potentially useful for patient education, often contain biased or suboptimal content, limiting their reliability as standalone resources[78, 109].

### Social Media in Neurosurgery: Advantages

The adoption of social media in neurosurgery has been accelerating, driven by its capacity to enhance communication, education, and collaboration on a global scale. Platforms such as X (formerly Twitter), Instagram, YouTube, and specialized medical forums have become valuable tools for the distribution of research, surgical techniques, and clinical cases[125]. These platforms offer borderless real-time exchanges of knowledge that were previously constrained by geographical and institutional barriers [107]. Neurosurgeons are now able to follow and contribute to the latest developments in the field, fostering a dynamic and collaborative environment [6, 85]. Furthermore, social media has been integrated into neurosurgical education and training [40, 76, 86]. Neurosurgical conferences nowadays often include live social media coverage, making discussions, presentations, and debates accessible to a broader audience, including those who may not be able to attend in person. For trainees, social media provides a platform for case-based learning, peer interaction, and the review of surgical videos and techniques that might not otherwise be readily available[8, 121]. This open sharing of knowledge has made neurosurgical education more accessible and created a more connected global neurosurgical community.

Additionally, content shared by neurosurgeons on social media platforms is often visible to the public, including patients, which adds another dimension to knowledge distribution. Patients may benefit from exposure to expert perspectives, improving their understanding of medical conditions and treatment options. Moreover, patient-generated content, such as personal experiences and health information shared on platforms like X (formerly Twitter) and Reddit, may provide additional valuable insights into patient health and perceptions, offering data that were previously difficult to access.

The observed rise in usage of social media platforms suggests that maintaining a social media presence can offer neurosurgeons various substantial benefits, including improved patient reviews, enhanced departmental reputation, and, in some cases, bolstered academic success. Moreover, social media may foster interdisciplinary collaboration, facilitate knowledge sharing, and improve access to educational content.

### Social Media in Neurosurgery: Challenges and Concerns

Despite the numerous benefits of social media diffusion in neurosurgery, its introduction also brings potential drawbacks that must be considered and addressed. One of the most significant challenges is the potential spread of misinformation. While social media allows for the rapid circulation of information, it also facilitates the unregulated sharing of non-evidence-based or incorrect medical advice[96]. Neurosurgeons must be cautious about the quality of content they engage with and share, ensuring that it aligns with evidence-based practices. Furthermore, patient confidentiality and privacy concerns are paramount, and any sharing of clinical cases must comply with ethical standards, including obtaining patient consent where appropriate[9, 39].

The legal implications of sharing medical content on social media, particularly in terms of intellectual property and patient confidentiality, can be complex[58]. Neurosurgeons must navigate these issues carefully, ensuring they are compliant with institutional and national regulations, as well as with the guidelines of professional societies. Furthermore, there is an ongoing debate about the role of social media in maintaining professional credibility[66, 91]. While one can successfully utilize social media platforms to enhance their reputations, there exists a risk of potentially blurring the lines between professionalism and self-promotion. Neurosurgeons must strike a balance between engaging in educational and collaborative exchanges and maintaining a professional demeanor that upholds the integrity of the field.

While challenges remain in content quality and bias, the strategic use of social media platforms holds immense potential for advancing neurosurgical practice, education, and research.

### Patient Information and Education

Patient education is a key strategy to mitigate low health literacy, which is strongly associated with adverse health outcomes[16, 102]. In neurosurgery, effective education is particularly important given the complexity of surgical procedures and the severity of the conditions treated. Social media platforms offer a unique opportunity to reach patients due to their widespread use. Among these, YouTube has emerged as a leading source of educational content. However, because such videos are not subject to peer review, studies included in this review have consistently reported limitations in their quality and reliability[13, 35, 47, 54, 80]. This is where the afore-mentioned suggestion for a peer-review process or curated repositories tailored to neurosurgery might prove valuable.

A further concern is that some videos may serve as disguised advertisements rather than objective educational material. Ward et al. highlighted this issue, noting that unlike traditional advertisements, such content often lacks clear labeling and may mislead viewers[122]. Similar challenges have been reported on platforms such as X (formerly Twitter) and Instagram, where paid promotional content is frequently posted without disclosure[122]. Addressing these issues is crucial to enhance the reliability of patient education materials in neurosurgery.

### Education of Healthcare Providers

Surgical education has undergone a profound transformation in recent decades with the introduction of virtual and augmented reality, high-fidelity simulators, and video-based platforms. More recently, social media has emerged as an additional avenue for surgical training[70]. Among these platforms, YouTube, Instagram, and X (formerly Twitter) have become the most widely used for disseminating educational content.

Evidence suggests that especially video-based learning can support technical skill acquisition among surgical trainees[3]. A study included in our review by Mayo et al. assessed the available YouTube training videos on external ventricular drain placement, one of the most common neurosurgical procedures. The authors found a large volume of videos available, but only few which were effective in regards to educational value and accessibility[76]. Similarly, Faisal et al. investigated the use of webinars for teaching cerebrovascular and endovascular procedures to physicians and trainees. Their findings indicate that webinar-based education was not only perceived as effective but also stimulated new research ideas, facilitating interaction across geographical boundaries and levels of training [41]. In this context, social media–based education holds particular promise for low- and middle-income countries, where it may enhance access to training resources, foster real-time collaboration, and enable case-based discussions[27, 40, 83]. Beyond education, these platforms may therefore play a broader role in advancing global neurosurgery.

### Collaboration

One of the most significant advantages of social media in neurosurgery is its ability to facilitate collaboration across diverse stakeholders, including academic institutions, journals, industry partners, clinics, and individual practitioners. These platforms provide opportunities for interactions that would otherwise be difficult to establish. An example of this is the “Neurosurgery Cocktail” group, which is currently the largest neurosurgical community on social media. With more than 30′000 followers worldwide, it serves as a community for sharing clinical experiences, announcing activities, and promoting collaboration among neurosurgeons across different regions[23, 67]. Such initiatives show how social media can generate meaningful impact for neurosurgeons worldwide.

### Research Dissemination and Academic Visibility

Another important advantage of social media lies in its ability to disseminate research, both from the perspective of individual authors and academic journals. For researchers, these platforms provide an opportunity to extend the reach of their work beyond traditional academic circles. Patel et al. demonstrated that authors’ social media activity was variably associated with altmetric metrics of visibility[90]. In contrast, studies evaluating systematic dissemination by journals revealed stronger effects, showing increases in article views, downloads, citations, and even positive influences on journal impact factors [57, 105, 120]. These findings support the establishment of an active social media presence by journals to maximize outreach and engagement.

Enhanced visibility on social media may also contribute to career advancement. Bandyopadhyay emphasized its potential to promote the inclusion of women in neurosurgery, highlighting how social media can be used to disseminate information, provide inspiration, mitigate biases, and facilitate mentorship[14]. Overall, these platforms enable networking and increase visibility and thus may potentially develop the path to new academic and professional opportunities.

### Limitations of the Study

The key limitation of this systematic review lies in its reliance on available publications, which may lead to underrepresentation of social media activity that is informal or unpublished. Additionally, the dynamic and rapidly evolving nature of social media implies that certain findings may lose relevance over time, particularly as new platforms emerge, and usage patterns shift. Furthermore, the reliance on language-specific sources may have introduced bias, potentially leading to an underrepresentation of platforms more commonly used in low- and middle-income countries.

### Future Perspectives

Neurosurgical institutions could significantly enhance the value of social media content by actively contributing and engaging with these platforms. Future efforts should focus on establishing clear guidelines for social media usage in neurosurgery, including standards for professionalism and patient confidentiality. Research should also explore the impact of emerging platforms and technologies, such as artificial intelligence-driven social media analytics, to enhance professional networking and knowledge sharing. Furthermore, studies on the impact of social media on patient outcomes, particularly in terms of understanding and satisfaction, are necessary to understand its broader implications in neurosurgical practice.

## Conclusion

Social media is an increasingly valuable tool in neurosurgery for education, patient engagement, collaboration, and research dissemination. Physicians should use it to share evidence-based information, while researchers and journals can enhance the visibility and impact of their work through active engagement. A deliberate, evidence-informed approach is necessary to harness the benefits of social media platforms for the field of neurosurgery.

## Supplementary Information

Below is the link to the electronic supplementary material.

ESM 1 (PDF 66.8 KB)

## Data Availability

The data in support of our findings can be obtained upon reasonable request from the corresponding author.
